# Fear of Sleep in Undergraduates with a History of Sexual Trauma

**DOI:** 10.3390/bs15111462

**Published:** 2025-10-27

**Authors:** Julia Russell, Favour Oloriegbe, Garrett Robert Baber, Anna K. Quesada, Nancy A. Hamilton

**Affiliations:** Department of Psychology, University of Kansas, 1415 Jayhawk Boulevard, Lawrence, KS 66045, USA; julia_russell@ku.edu (J.R.); garrett.baber@ku.edu (G.R.B.); aquesada@ku.edu (A.K.Q.)

**Keywords:** fear of sleep, psychological trauma, nightmares, posttraumatic stress disorder

## Abstract

Fear of sleep may drive the development of trauma-related sleep disturbances but may differ across potentially traumatic events (PTEs). This study tested whether the factor structure of the Fear of Sleep Inventory-Short Form (FOSI-SF) differed between women with a history of sexual traumas (ST: including sexual assault and other sexual traumas) and women reporting other Non-ST PTEs. Two samples of undergraduate women who endorsed a history of PTEs (*n* = 339 and *n* = 318) completed a battery of questionnaires including the FOSI-SF, as well as other psychological and sleep screening measures. We conducted an exploratory factor analysis in Sample 1 and a confirmatory analysis in Sample 2. In the sample of women endorsing a history of ST, but not those with Non-ST PTEs, four latent factors adequately fit the data: vigilance, fear of the dark, fear of nightmares, and vulnerability. This structure was replicated in the second sample that included only women with a history of ST and showed appropriate convergent and discriminant validity with other study measures. This study illustrates that fear of sleep may differ across PTE, and that for those with a history of ST, the fear of nightmares appears to be distinct from other facets of the fear of sleep construct.

## 1. Introduction

Sexual violence is a disturbingly common phenomenon with potential medical and psychological consequences, including sleep problems. Women are 2–3 times more likely to experience sexual assault than men, and decades of research shows that sexual trauma (ST) is associated with subsequent sleep problems, particularly for women (Gallegos et al., 2021). Sleep problems, such as nightmares and insomnia, are common symptoms of Post-traumatic Stress Disorder (PTSD) but are not exclusive to those with a PTSD diagnosis (Milanak et al., 2019). As such, it is critical to understand psychological processes that contribute to the development and maintenance of sleep disturbances following PTE.

Sleep disturbances have been associated with exposure to potentially traumatic events (PTEs) in both clinical and non-clinical populations (Pruiksma et al., 2014). However, the impact on sleep varies across PTEs. For instance, reports of a history of sexual assault have been found to have a stronger relationship with sleep complaints than other Non-ST PTEs such as car accidents (Lind et al., 2017; Steine et al., 2012a). A history of sexual assault and sexual harassment were associated with worse sleep quality in a sample of midlife women and sample of flight attendants (Gale et al., 2019; Reynaud et al., 2023; Thurston et al., 2019). Among a large sample of Norwegian college students, a history of ST (e.g., sexual assault, sexual harassment) was linked to sleep disturbances (Steine et al., 2021b). Compared with students without an assault history, students with a history of sexual assault were at 6.12 times the risk of weekly nightmares (terrifying dreams that wake you up and make it difficult to return to sleep) and 2.48 times more likely to meet the insomnia criteria, meaning difficulty initiating or maintaining sleep. Similarly, students reporting other sexual trauma including sexual comments, looks or gestures, or photographs had more than twice the risk of weekly nightmares. Germaine to the current study, the relationship between PTEs and insomnia was found to be mediated by nightmares, suggesting perhaps that anticipation (or fear of) nightmares may drive insomnia. These data are consistent with a recent theoretical paper that proposed that nightmares are a key driver of fear of sleep and that fear of sleep is an underlying mechanism linking trauma exposure to insomnia (Werner et al., 2021).

Although many PTEs including ST have been found to be associated with insomnia symptoms and nightmares, there has been relatively less research designed to identify mechanisms driving individual sleep problems or pathways by which sleep problems are connected; however, a recent theoretical paper is a noteworthy exception, proposing that the fear of sleep plays a key role in linking trauma exposure to insomnia (Werner et al., 2021). It was proposed that the fear of sleep is driven by PTSD symptomatology: safety fears, vigilance, loss of control, and nightmares and that the avoidance drives maladaptive sleep behaviors. This model is strongly supported by data showing that the fear of sleep partially accounted for the association between sexual assault and insomnia in young African American adults (Hall Brown et al., 2015). Fear of sleep may also be a marker of clinical severity. Compared with undergraduates, most of whom reported experiencing at least one PTE, clinical patients with a history of PTE and chronic nightmares reported higher levels of fear of sleep. Fear of sleep also appears to be closely linked to trauma-related nightmares. Participants with chronic or acute nightmares reported higher levels of fear of going to sleep than people without nightmares (Drexl et al., 2019). As such, this model and the data suggest that the fear of sleep is a multidimensional construct linking nightmares to insomnia.

Fear of sleep is most comprehensively measured by the Fear of Sleep Inventory (FoSI) (Zayfert et al., 2006). The original version of the FOSI included 23 items, with items measuring the frequency of fear of sleep driven by causes including fear of the dark, fear of nightmares, and vigilance (attempts to maintain alertness to threats). The original scale was validated in a sample of urban dwelling African American young adults, most with exposure to trauma. Five factors were derived from the data: fear of sleep, fear of loss of vigilance, fear of re-experiencing trauma (nightmares), vigilant behaviors, and fear of the dark (Huntley et al., 2014). This factor structure hewed most closely to the model proposed by Werner (Werner et al., 2021). However, in subsequent research, versions of the FOSI have varied in terms of the number of items, and the original factor structure has failed to be replicated. In a sample of undergraduates with an undefined trauma history, the original 23-item FoSI initially yielded an unclear factor structure and was reduced to 13 items. The 13 item FoSI-short form (FoSI-SF) was found to have a two-factor structure (fear of loss of control and fear of darkness). The two-factor structure of the FoSI-SF was then replicated and found to have adequate measurement properties in a second sample of 67 patients with a variety of past PTEs and the total score was found to correlate with PTSD symptoms and insomnia symptoms (Pruiksma et al., 2014). The factor structure of the FoSI-SF was again examined in a German sample with translation, but the authors did not finalize a factor structure due to statistical Heywood cases (Drexl et al., 2019). To our knowledge, these studies form the basis of the small psychometric literature surrounding the FoSI-SF.

Werner and colleagues summarized their theoretical model of fear of sleep as follows: the fear of sleep is driven by two factors, that of safety concerns/loss of control and fear of nightmares (Werner et al., 2021). However, factor structures of the FOSI have largely failed to show distinct nightmare and safety concern/loss of control factors. One possible explanation for this discrepancy is that the components of the fear of sleep may vary between people who have been exposed to different PTEs and may indeed have a different factor structure across populations. While multiple PTEs are associated with insomnia symptoms, there may be different mechanisms precipitating or maintaining sleep problems following ST compared to other PTEs such as motor vehicle accidents. For example, following ST, women may be more attuned to cues related to safety in a bed if it mimics the setting of assault or aspects of other sexual trauma. Over one third of female rape survivors reported being assaulted by an intimate partner (Basile et al., 2022), violating feelings of safety in personal spaces such as the home or bedroom. Relatedly, the strong association of ST with nightmares (Steine et al., 2021b) may also influence the factor structure, both in terms of factor loadings as well as the relationship to other factors. Conversely, for patients who develop a fear of sleep following a car accident, vulnerability surrounding the sleep environment would be less likely to be a concern and nightmares are less common following car accidents (Lind et al., 2017; Steine et al., 2012a). Thus, the environmental context during different PTEs may lead to different cognitions, behaviors, and emotional reactions to the sleep routine and setting, affecting the mean level differences for the components of fear of sleep and/or resulting in different factor structures.

To summarize, the extant literature has demonstrated a strong link between trauma and sleep problems such as insomnia and nightmares, and a recent theoretical model has identified the fear of sleep as a key driver of insomnia following trauma (Werner et al., 2021). However, the factor structure of the FoSI-SF remains unclear and does not map onto theoretical antecedents of the fear of sleep. One possible explanation for the mismatch is that the latent, multidimensional fear of sleep construct may vary across PTEs. For instance, exposure to ST appears to be associated with high rates of nightmares, whereas accidental traumas may not (Lind et al., 2017; Steine et al., 2021b). Those mean level differences may also result in differences in variance shared by items of the FOSI-SF, leading to a different factor structure. Following this assumption, we hypothesized that the FOSI-SF factor structure would vary by PTE type. Although we did not make specific predictions about the number of factors for those with ST and those with other Non-ST PTEs, we expected that a fear of nightmares factor would emerge in those with a history of ST. As such, the goal of this study was to test the factor structure of the FoSI-SF in undergraduate students, comparing college-aged women who endorsed a history of ST with those who had a history of other types of Non-ST PTEs.

## 2. Materials and Methods

### 2.1. Participants

Participants were young adult female undergraduate students from a large Midwestern university enrolled in an introductory psychology class. Two samples were collected: Sample 1 was collected during the 2021 academic year, and Sample 2 was collected during the 2022 academic year. Individuals over the age of 24 (*n* = 12 in both samples) were removed from the dataset to reflect typical college student ages. Women who did not endorse experiencing any PTEs (*n* = 25 and *n* = 152, respectively) were removed from the dataset with the goal of comparing the FOSI across exposures to different PTEs. Participants were divided into two groups: (1) ST—those who endorsed past STs (sexual assault or other sexual traumas) and (2) Non-ST PTEs—those who endorsed PTE experiences that were not ST. The most commonly endorsed PTE in the Non-ST PTE was an automobile accident.

### 2.2. Procedure

Data from both samples were gathered using a similar procedure. Participants were directed to a link via an online research pool system and reviewed details of informed consent before completing surveys. The battery of questionnaires for both samples included the FOSI but were not identical across groups. Participants received course credit for completing the following battery of questionnaires for partial fulfillment of course requirements.

### 2.3. Measures

#### 2.3.1. Demographics (Samples 1 and 2)

A five-item questionnaire was used to assess demographic information. The questionnaire asked participants to report their age, gender identity, marital status, race/ethnicity, and sexual orientation. Participants were able to decline identifying or select multiple options.

#### 2.3.2. Life Events Checklist (LEC: Samples 1 and 2)

The Life Events Checklist (LEC-5) is a 16-item self-report questionnaire designed to assess exposure to PTEs (Weathers et al., 2013a). The survey uses a 6-point nominal scale to assess methods of trauma exposure (happened to me, witnessed it, learned about it, part of my job, not sure, or does not apply). In this study, exposure to ST was defined as endorsing “happened to me” for either or both of two items: item 8—sexual assault (rape, attempted rape, and made to perform any type of sexual act through force or threat of harm), and item 9—other unwanted or uncomfortable sexual experience. We scored items where participants endorsed that a PTE “happened to me” as 1 and other responses as 0. A previous version of the checklist was shown to have adequate convergent validity with other measures of PTE exposure in both an undergraduate and veteran sample (Gray et al., 2004).

#### 2.3.3. Fear of Sleep Inventory-Short Form (FOSI-SF; Samples 1 and 2)

The Fear of Sleep Inventory 13-Item Short Form (FOSI-SF) uses a 5-point Likert scale to assess self-reported frequency of fear related to sleep and nighttime experiences (not at all, a few times a month, once or twice a week, several times per week, and nearly every night). An example item is, “I woke up in the night, and I was terrified of returning to sleep.” Cronbach’s α = 0.90 for the FoSI SF, suggesting good internal consistency (Pruiksma et al., 2014).

#### 2.3.4. Pittsburgh Sleep Quality Index (PSQI, Sample 1)

The PSQI was used to assess sleep quality and sleep disturbances over a one-month interval. The 19 items produce seven component scores: subjective sleep quality, sleep latency, sleep duration, habitual sleep efficiency, sleep disturbances, use of sleeping medication, and daytime dysfunction. The summation of these component scores yields a global score which ranges from 0 to 21, with higher scores indicative of worse sleep. Cronbach’s α = 0.83 for the PSQI, suggesting good internal consistency (Buysse et al., 1989).

#### 2.3.5. Patient Health Questionnaire (PHQ-8 and PHQ-9, Samples 1 and 2)

The PHQ-8 is an eight-item questionnaire that assesses the depressive symptoms of individuals over the prior two-week period (not at all, several days, more than half the days, and nearly every day); the PHQ-9 includes the same items with an additional item (9) assessing suicidality. An example item is, “feeling down, depressed,, or hopeless.” Scores range from 0 to 24 or 0 to 27 points, with suggested clinical thresholds being no significant depressive symptoms (0–4), mild depressive symptoms (5–9), moderate symptoms (10–14), moderate–severe (15–19), and severe symptoms (20 and above) (Kroenke et al., 2001).

#### 2.3.6. Nightmare Disorder Index (NDI, Sample 2)

The Nightmare Disorder Index (NDI) is a brief self-report screening measure for symptoms related to Nightmare Disorder (Dietch et al., 2021). Items related to nightmare frequency, distress, impairment, and duration are rated on a 0–4 scale. Items can be scored dichotomously to assess congruence with diagnostic Nightmare Disorder criteria, or a continuous score (range 0–20) can be produced, with higher scores indicating more severe nightmares. When used to produce a continuous score, internal consistency has been shown to be in the good range, with Cronbach’s α = 0.80.

#### 2.3.7. Pre-Sleep Arousal Scale (PSAS: Samples 1 and 2)

The Pre-Sleep Arousal Scale (PSAS) is a self-report measure of states of arousal prior to sleep onset. The scale was originally tested in two groups of college students, both with those endorsing insomnia symptoms and those with normative sleep. Items are summed and produce two scales, cognitive and somatic, with higher scores on both indicating higher levels of arousal. Internal consistency for these subscales range from acceptable to good (α = 0.67 to α = 0.88 for the cognitive subscale; α = 0.79 to α = 0.84 for the somatic subscale) (Nicassio et al., 1985).

#### 2.3.8. PTSD Checklist for DSM-5 (PCL-5, Sample 2)

The PTSD Checklist for DSM-5 (PCL-5) is a 20-item self-report measure assessing DSM-5 symptom clusters in PTSD in the past month in relation to the self-reported worst event (Weathers et al., 2013b). The self-report scale is rated 0–4 for each item (not at all–extremely); a total score can be calculated (range 0–80) or symptom clusters can be scored (Weathers et al., 2013b). An example item is “Feeling very upset when something reminded you of the stressful experience.” In undergraduate samples, a clinical cutoff score of 38 indicating possible PTSD was established (Cohen et al., 2014), and Cronbach’s α = 0.94 for the PCL-5, suggesting excellent internal consistency (Blevins et al., 2015).

### 2.4. Data Analysis

To establish whether previously used factor structures for the FOSI-SF were adequate and equivalent across samples, we began by using Confirmatory Factor Analysis (CFA) to estimate the factor structure of FOSI-SF in Sample 1, separately by group (ST vs. Non-ST PTE). Guided by prior psychometric studies (Pruiksma et al., 2014), we tested one- and two-factor models and tested for configural invariance. Because of poor model fit in the ST group in Sample 1 for both one and two-factor models, we opted to conduct Exploratory Factor Analyses (EFA) with data from Sample 1. EFA models were estimated using robust Diagonal Weighted Least Squares, because the FOSI-SF contains Likert items. Parallel analysis was used initially to suggest the optimal number of factors after plotting eigenvalues of simulated data in comparison with the observed data (Horn, 1965). Factor analytic fit statistics were selected from commonly used metrics of fit and used commonly employed cutoffs: the Standardized Root Mean Square Residual (SRMR) < 0.08 and the Comparative Fit Index (CFI) > 0.90, with CFI > 0.95 denoting excellent fit. Root Mean Square Error of Approximation (RMSEA) was also used; RMSEA values < 0.05 are considered good, with values between 0.05 and 0.08 acceptable (Hu & Bentler, 1999). After conducting an EFA in Sample 1 to determine a factor structure for the ST group, we conducted a confirmatory analysis (CFA) of this model in Sample 2. Analyses were conducted in RStudio version 4.0.5 with packages lavaan and psych used for primary analyses (Posit Team, 2025; Revelle, 2022; Rosseel, 2012). Pearson’s product–moment correlations were used to provide correlations of variables in this analysis, and confidence intervals of the correlations between factor scores and observed variables were compared to assess for differences (Zou, 2007).

## 3. Results

Sample characteristics for both Sample 1 and Sample 2 are presented in Table 1 and Table 2. Table 1 lists characteristics of the participants endorsing ST (*n* = 204) compared to the Non-ST PTE group (*n* = 135). The median number of PTE experienced by both Sample 1 and Sample 2 was two events in their lifetimes. The proportion of participants in both samples endorsing each PTE on the LEC can be found in Supplementary Table S1. In Sample 1, individuals in the ST group endorsed a greater number of symptoms of depression, and more problems with general sleep disturbance (PSQI) and fear of sleep compared to the Non-ST PTE group. Similarly, in Sample 2, individuals who endorsed ST reported greater problems with depression, fear of sleep, pre-sleep arousal, PTSD symptoms, and nightmares.

The ST groups in Samples 1 and 2 differed on some measures. Sample 2 endorsed significantly more PTEs than Sample 1 (mean = 3.61 for Sample 1, mean = 3.07 for Sample 2, *p* = 0.004) and higher levels of somatic pre-sleep arousal (PSAS somatic subscale mean = 15.92 in Sample 1, PSAS somatic subscale mean = 17.46 in Sample 2, and *p* = 0.03). Sample 1 endorsed a higher level of fear of sleep on the FOSI-SF (Sample 1 mean = 18.21, Sample 2 = 8.14, and *p* < 0.0001). There were no other between-group differences.

Results of factor analyses are presented in Table 3. A CFA with single and two factors resulted in a poor fit for Sample 1. Initial steps to establish configural invariance included fitting a CFA separately within each group. The CFA resulted in a worse fit in the ST group than the Non-ST PTE group, suggesting a possible misspecification in the ST group. We also attempted a multigroup model, but the CFI fell below the acceptable level, and the SRMR was borderline—suggesting room for improvement with the model fit. Given the apparent disparity in model fit between the ST and Non-ST PTE groups, an exploratory analysis was undertaken to describe the factor structure in the ST group within Sample 1.

Parallel analysis suggested four factors would adequately explain the observed data in the ST group in Sample 1. We pursued EFA for four factors with oblimin rotation, which allowed for correlated factors. This process produced the factor structure described in Table 4, with four factors explaining 62% of the total variance in scores for Sample 1. The final factor structure resulted in four factors: vigilance, fear of the dark, fear of nightmares, and vulnerability. Items that loaded on the vigilance factor appeared related to the pre-sleep experience (i.e., while in bed), while items loading on vulnerability more generally described the loss of safety and autonomy while falling or being asleep. Items that loaded onto the fear of nightmares factor described fear of nightmares and items loading on fear of the dark face a validly described fear of the dark. Each factor produced moderate to large correlations with other observed outcomes including general sleep quality (PSQI), sleep hygiene, emotion regulation, and depression symptoms.

The model produced by the EFA was next tested using CFA on an additional sample of undergraduate students (Sample 2) who participated in data collection approximately one year after the initial sample. The four-factor structure yielded an adequate fit in the ST group (Table 3). When the four-factor model was tested on the Non-ST PTEs group from Sample 2, the model demonstrated extremely high covariance between the three latent factors—vigilance, fear of nightmares, and vulnerability—suggesting that items would load best on a single factor and that the four-factor model did not perform well with this group. This suggests that our four-factor model was unique to the ST group. In the four-factor model with the ST group, modification indices suggested two items (I was aware of being especially vulnerable when I am asleep and I stayed up late to avoid sleeping) cross-loaded on two factors. Given the possibility of overfitting the sample by removing items in this manner, our final presented model includes four factors with all 13 FOSI-SF items. Table 4 shows the standardized pattern coefficients of the four-factor model in Sample 2 with all items included; higher pattern coefficients suggest a stronger loading of the indicator onto the latent variable.

We also examined the correlations between factor scores and other variables measured in the replication sample of women who had been exposed to ST (Table 5). All factors’ correlations with PTSD symptoms, pre-sleep arousal, and correlations with nightmares were generally stronger than those of depression and insomnia symptoms. For the nightmare factor, correlations were strongest with the PCL, somatic PSAS, and the NDI. The vulnerability factor showed the strongest correlations with the PCL and somatic PSAS subscale. The vigilance factor showed the strongest correlations with the PCL and somatic PSAS subscale, while fear of the dark had relatively similar correlations across the somatic and cognitive PSASs and the PCL. Factors for vigilance and fear of the dark showed weaker correlations with the NDI. Across all factors, correlations were small with the PHQ-9 and moderate with the ISI. FoSI-SF factors were most strongly related to PTSD symptoms and pre-sleep arousal, and factors related to vulnerability and nightmares were more closely related to Nightmare Disorder symptoms than other factors.

## 4. Discussion

In this study, we tested the factor structure of the FOSISF in women who reported a history of ST compared to women who reported Non-ST PTEs like car accidents. In contrast to the previously derived two-factor structure, a four-factor structure emerged that included factors labeled as vigilance, fear of the dark, fear of nightmares, and vulnerability. These factors closely map onto the theoretical antecedents and behaviors associated with fear of sleep laid out by Werner et al. (2021). This factor structure represented the best fit for the sample of women with ST but was poor fit in a sample of women who endorsed Non-ST PTEs. Factors in the four-factor structure showed the strongest correlations with other measures related to disturbed sleep, including pre-sleep arousal and PTSD symptoms. Items related to vulnerability and nightmares were most strongly related to Nightmare Disorder symptoms. Correlations suggested closely related yet distinct constructs that showed relationships with different aspects of trauma-related symptoms and sleep disturbance. The factor structure was also distinct from insomnia and depressive symptoms, demonstrating discriminant validity.

The FOSI-SF demonstrated a reliable factor structure in our two samples with ST history, but the factor structure differed across PTE types such that the fear of sleep in women who endorsed ST appeared to be best accounted for by four rather than two latent variables, and none of the factor structures were replicated in the sample of women who had not experienced ST. It was noteworthy that a separate factor encompassing items related to nightmares emerged in the sample of women who reported ST. This is consistent with the body of literature showing a high prevalence of trauma-related nightmares among those exposed to sexual violence (Gallegos et al., 2021). For instance, in an undergraduate sample, women reporting a history of sexual or physical abuse as well as neglect reported higher nightmare frequency than those who did not endorse abuse or neglect; they also reported higher levels of nightmare distress in comparison to women who had experienced psychological abuse and emotional neglect (Duval et al., 2013). Nightmares also appear to be intertwined with insomnia symptoms. In the sample of Norwegian students, experiences of sexual harassment and assault were associated with increased odds of insomnia symptoms, with the effect partially mediated by nightmare frequency (Steine et al., 2021b). Consistent with this study, the fear of nightmares emerged as a distinct factor in students who reported ST. Although we posit that there is something distinct about ST and the fear of sleep, additional comparisons are warranted examining the FOSI factor structure in other trauma-exposed populations.

While the 13 items of the FOSI-SF showed evidence of high internal consistency, it is worth further exploring why the underlying structure of the measure differed across samples reporting different types of PTEs. We propose that the characteristics of PTEs may play a role in subsequent sleep disturbances. It may be that experiences of ST, which have associations with the bedroom environment, increased distress related to sleep and bed that was different than for those experiencing other types of PTEs. Consistent with this explanation, interpersonal PTEs (including sexual assault), but not accidental PTEs (e.g., vehicle accidents), predicted sleep disturbances as measured by a 4-item PSQI (Lind et al., 2017). Conversely, another study found that including the PTE type in models predicting sleep disturbance was not more precise than including a total number of past PTEs (Milanak et al., 2019). In our first sample, the ST group endorsed more PTEs than the Non-ST PTE group, so it is impossible to rule out in this analysis that the number of PTEs could be related to factor structure in that sample. However, our factor structure showed adequate fit across the two samples with ST despite differences in number of PTEs and FoSI-SF scores.

One of the clear advantages of the four-factor structure derived from these data is that it facilitates tests of theoretical models related to the fear of sleep. In particular, the four factors we derived fit the model proposed by Werner et al. (2021). Mapping on to that model, we propose that PTSD avoidance drives the FOSI fear of the dark cluster, whereas negative beliefs about safety drive FOSI vulnerability and vigilance. Building on that model and incorporating additional work about nightmares (Youngren et al., 2024a, 2024b), we propose that the FOSI nightmare cluster is driven by frequent nightmares and perpetuated by autonomic hyperarousal. As such, the four-factor FOSI model would add additional clarity to theories linking nightmares to insomnia.

Although this study brings new information regarding the latent factors underlying fear of sleep and how these factors vary by PTE, there were important limitations. Sample sizes for the exploratory analyses were relatively small, thus additional confirmatory analyses in other samples are warranted. Future studies should consider using Item Response Theory (IRT) to determine the optimal number of items for each factor and with a goal of reducing the burden on the reporting individual. Additional research would benefit from using measures above and beyond screening questionnaires for psychological concerns to clarify clinically significant symptoms that may contribute to sleep disturbances. This study included only female undergraduate students, and future research should include community members, particularly non-student women aged 18–24, who are in fact more likely to experience SA than women of the same age who are students (Sinozich & Langton, 2014). Even so, campus sexual violence statistics are strikingly high, as 25.9% of undergraduate women reported nonconsensual sexual contact by physical force or inability to consent, and 31.3% of undergraduate women reported experiencing harassment that interfered with their ability to participate or created a hostile environment during the period since enrollment (Cantor et al., 2015). Finally, from a theoretical perspective, longitudinal data would help to clarify the trajectory of the relationships between the fear of sleep and other sleep problems such as insomnia and nightmares. Although it makes intuitive sense that nightmares would predict insomnia and that the relationship would be mediated by fear of sleep, these cross-sectional correlational data do not rule out other causal trajectories or that a third variable could be the main driver of all these relationships.

Adequate psychometric analysis is necessary to assess available measures and to make meaningful comparisons. This study examined the factor structure of the FoSI-SF across different PTEs, grouped by ST events. This analysis showed that women with a history of ST experienced higher levels of fear of sleep than those reporting other types of PTEs (Non-ST PTE group). Additionally, the factor structure of the measures appeared to differ such that a differential pattern of common factors contributed to overall levels of fear of sleep within the sample. Our analysis produced a revised factor structure of the FoSI-SF women with a history of ST that may offer greater utility to trauma researchers than the extant two-factor model. Each of the factors could be used as subscales (with adequate internal consistency) to separately assess the fear of sleep driven by vigilance, fear of the dark, fear of nightmares, and feelings of vulnerability.

## 5. Clinical Significance

The utility of a measure’s factor structure is determined not only by replicability but by whether it is useful for research and clinical practice. A clear factor structure of the FoSI has direct relevance for those providing clinical services to women with an ST history who are reporting trouble sleeping. Implications of elevation on factors such as vigilance and vulnerability are distinct from elevation on the nightmare factor and should be considered in the patient’s environmental context. For instance, reducing safety behaviors is an important focus of cognitive behavioral therapy for insomnia (CBTi) and in treatments for PTSD (Lancee et al., 2015; Harvey, 2002). However, we posit that, in the cases where there are true threats to safety, such as in communal living and stalking (Buhi et al., 2009), safety changes (e.g., more secure locks) may be helpful. By facilitating these structural safety changes, a patient may feel more inclined to engage in therapy to subsequently target dysfunctional beliefs around the safety of their sleeping environment. In contrast, fear of nightmares would likely be best addressed using clinical interventions that target nightmares and nightmare-related arousal, such as image rehearsal therapy (IRT) or exposure, relaxation, and rescripting therapy (ERRT) focused specifically on nightmares (Davis et al., 2011; Krakow et al., 2001). However, these clinical distinctions would be lost when relying on a single fear of sleep summary score or extant two-factor models.

## 6. Conclusions

The findings of this study are consistent with the previous literature indicating that women experience significant distresses following ST, with specific struggles in sleep disturbances. Women are generally at a higher risk of experiencing ST and college students are more so. Among those who experienced sexual trauma—rape, attempted rape, made to perform any type of sexual act through force or threat of harm, or any other sexual trauma—a four-factor latent structure emerged for the fear of sleep: vigilance, fear of the dark, fear of nightmares, and vulnerability. Additionally, these results differed by PTE. Although a two-factor structure has been evident in the past, we believe that further differentiating factors could paint a more specific picture of the nature of sleep disruption among those who have been exposed to sexual trauma. By continuing to investigate sexual assault, we hope that, in turn, survivors may improve sleep and mental health, therefore reclaiming their autonomy.

## Figures and Tables

**Table 1:** Characteristics of Sample 1.

	ST (*n* = 204)	Non-ST PTE (*n* = 135)	Overall (*N* = 339)	Cohen’s *d*
Age (Years)	18.6 (0.92)	18.5 (0.80)	18.5 (0.87)	
Ethnicity				
American Indian or Alaska Native	1 (0.50%)	0 (0%)	1 (0.3%)	
Asian	8 (3.90%)	12 (8.90%)	20 (5.9%)	
Black or African American	12 (5.90%)	7 (5.20%)	19 (5.6%)	
Hispanic or Latinx	14 (6.90%)	5 (3.70%)	19 (5.6%)	
Middle Eastern or North African	2 (1.0%)	2 (1.50%)	4 (1.2%)	
Native Hawaiian or Other Pacific Islander	1 (0.5%)	0 (0%)	1 (0.3%)	
White	148 (72.5%)	94 (69.60%)	242 (71.4%)	
Endorsed Multiple Identities	16 (7.8%)	13 (9.60%)	29 (8.6%)	
Did Not Self-Report	2 (1.0%)	2 (1.50%)	4 (1.2%)	
Gender				
Cisgender Female	197 (96.60%)	131 (97.00%)	328 (96.8%)	
Non-binary/Genderqueer	3 (1.50%)	2 (1.5%)	1 (0.3%)	
Endorsed Multiple Identities	1 (0.50%)	1 (0.7%)	2 (0.6%)	
Did Not self-identify	1 (0.50%)	0 (0.0%)	5 (1.5%)	
Patient Health Questionnaire-9 *	10.5 (5.91)	7.27 (5.18)	9.22 (5.84)	0.57
Fear of Sleep Index *	18.2 (15.20)	11.8 (11.8)	15.7 (14.3)	0.46
Pittsburgh Sleep Quality Index *	8.14 (3.70)	6.59 (3.16)	7.51 (3.56)	0.45
Pre-Sleep Arousal (Cognitive) *	24.3 (8.73)	20.9 (7.85)	23.0 (8.55)	0.40
Pre-Sleep Arousal (Somatic) *	15.9 (6.93)	13.6 (5.68)	15.0 (6.55)	0.36
Sleep Hygiene Index *	24.3 (8.49)	21.6 (7.54)	23.2 (8.22)	0.33

* indicates significantly different at *p* < 0.5.

**Table 2:** Characteristics of Sample 2.

	ST *n* = 175	Non-ST PTE *n* = 143	Overall *N* = 318	Cohen’s *d*
Age (Years)	18.47 (0.71)	18.49 (0.75)	18.5 (0.73)	
Ethnicity				
Asian	8 (4.6%)	5 (3.5%)	13 (4.1%)	
Black or African American	4 (2.3%)	8 (5.6%)	12 (3.8%)	
Hispanic or Latinx	12 (6.9%)	6 (4.2%)	18 (5.7%)	
Middle Eastern or North African	3 (1.7%)	0 (0%)	3 (0.9%)	
White	125 (71.4%)	102 (71.3%)	227 (71.4%)	
American Indian Alaska Native	0 (0%)	3 (2.1%)	3 (0.9%)	
Multiethnic	23 (13.1%)	18 (12.6%)	41 (21.9%)	
Gender-Female	175 (100%)	143 (100%)	318 (100%)	
Patient Health Questionnaire-9 *	10.39 (6.69)	6.08 (5.47)	8.43 (6.52)	0.70
Fear of Sleep Index *	8.14 (9.52)	3.41 (5.39)	5.97 (8.23)	0.60
Pre-sleep Arousal Scale (Cognitive) *	25.22 (7.95)	20.74 (8.25)	23.2 (8.38)	0.55
Pre-sleep Arousal Scale (Somatic) *	17.46 (6.81)	13.99 (5.75)	15.9 (6.57)	0.55
PTSD Checklist for DSM *	44.99 (16.2)	33.71 (13.42)	39.9 (16.0)	0.75
Nightmare Disorder Index *	8.45 (3.87)	6.58 (2.99)	7.75 (3.67)	0.53

* indicates significantly different at *p* < 0.5.

**Table 3:** Fit statistics of exploratory and confirmatory models.

Sample	Model Type	Chi Square	Degrees of Freedom	CFI	SRMR	RMSEA
Sample 1	Single-factor Model	181.11	65	0.66	0.08	0.07
	Two-factor Model, ST group ^a^	132.31	64	0.69	0.08	0.07
	Two-factor Model, Non-ST PTE group ^a^	73.168	64	0.92	0.09	0.03
	Multigroup Model	197.128	128	0.79	0.08	0.06
	Four-factor Model, ST Group	90.19	59	0.86	0.06	0.05
Sample 2	Four-factor model, ST group	83.99	59	0.91	0.05	0.06

Fit statistics for exploratory and confirmatory models. ^a^ Two-factor model detailed in Pruiksma and colleagues. CFI = Comparative Fit Index; SRMR = Standardized Root Mean Square Residual; and RMSEA = Root Mean Square Error of Approximation.

**Table 4:** Final factor loadings; confirmatory sample of women with exposure to ST (*n* = 175).

Item Content	Vigilance	Fear of the Dark	Fear of Nightmares	Vulnerability
I was afraid to close my eyes.	0.85			
I felt that it was dangerous to fall asleep.	0.77			
I tried to stay as alert as I could while lying in bed.	0.84			
I tried to stay alert to any strange noises while going to sleep.	0.74			
I was aware of being especially vulnerable when I am asleep.		0.86		
Being in the dark scared me.		0.80		
I slept with a light on to feel safer.		0.66		
I woke up in the night, and I was terrified of returning to sleep.			0.82	
I avoided going to sleep because I thought I would have really bad dreams.			0.76	
I awoke in the middle of the night from a nightmare and avoided returning to sleep because I might go back into the nightmare.			0.65	
I stayed up late to avoid sleeping.			0.54	
I was fearful of letting my guard down while sleeping.				0.72
I was fearful of the loss of control that I experience during sleep.				0.83

Item loadings on four-factor latent structure of Fear of Sleep Inventory-Short Form with study replication sample of women with exposure to ST.

**Table 5:** FOSI SF: Descriptives and correlations in a sample of college-aged women with a history of ST (*n* = 175).

FOSI SF and Subscales	M (*SD*)	α	PHQ-9	PCL	PSA-C	PSA-S	NDI	ISI
Vigilance (F1)	5.67 (2.87)	0.852	0.23	0.47	0.40	0.47	0.39	0.31
Fear of the Dark (F2)	5.61 (3.34)	0.80	0.24	0.43	0.41	0.45	0.31	0.31
Fear of Nightmares (F3)	6.81 (3.25)	0.81	0.27	0.54	0.44	0.53	0.51	0.36
Vulnerability (F4)	3.12 (1.84)	0.769	0.29	0.53	0.44	0.56	0.46	0.37
Total FOSISF	21.22 (9.54)	0.90	0.30	0.54	0.48	0.54	0.45	0.38

Pearson correlations between factor scores and observed variables in Sample 2, a sample of women who were exposed to ST. *Note*: All correlations significant at *p* < 0.05. FoSI SF = Fear of Sleep Inventory-Short Form. PSA-C = Pre-sleep Arousal–Cognitive. PSA-S = Pre-sleep Arousal–Somatic. NDI = Nightmare Disorder Index. ISI = Insomnia Severity Index.

## Data Availability

The data presented in this study are available on request from the corresponding author. The data are not publicly available because of concerns for maintaining privacy given the sensitive nature of the data.

## Supplementary Materials

The following supporting information can be downloaded at: https://www.mdpi.com/article/10.3390/bs15111462/s1, Supplementary Table S1. Proportion of endorsement of experiencing each Potentially Traumatic Event of the Life Events Checklist in Samples 1 and 2.

## Abbreviations

The following abbreviations are used in this manuscript:

CFA	Confirmatory Factor Analysis
CBTi	Cognitive Behavioral Therapy for Insomnia
CFI	Comparative Fit Index
DSM-5	Diagnostic and Statistical Manual of Mental Disorders, Fifth Edition
EFA	Exploratory Factor Analysis
ERRT	Exposure, Relaxation, and Rescripting Therapy
FoSI	Fear of Sleep Inventory
FoSI-SF	Fear of Sleep Inventory-Short Form
IRT	Image Rehearsal Therapy
ISI	Insomnia Severity Checklist
LEC	Life Events Checklist
N	Total Sample Size
n	Subgroup Sample Size
NDI	Nightmare Disorder Index
PCL-5	PTSD Checklist for DSM-5
PHQ	Patient Health Questionnaire
PTE	Potentially Traumatic Events
PTSD	Posttraumatic Stress Disorder
PSAS	Presleep Arousal Scale
PSA-C	Presleep Arousal Scale, Cognitive Subscale
PSA-S	Presleep Arousal Scale, Somatic subscale
PSQI	Pittsburgh Sleep Quality Index
r	Pearson Correlation Coefficient
RMSEA	Root Mean Square Error of Approximation
SA	Sexual Assault
SD	Standard Deviation
SHI	Sleep Hygiene Index
SHAPS	Sleep Hygiene Awareness and Practice Scale
SHST	Sleep Hygiene Self Test
SRMR	Standardized Root Mean Square Residual
ST	Sexual Trauma
α	Cronbach’s Alpha
