# Supplementary material for: Fear of Sleep in Undergraduates with a History of Sexual Trauma
Source: Behav Sci (Basel). 2025 Oct 27;15(11):1462. doi: 10.3390/bs15111462 (PMC12649145; doi:10.3390/bs15111462)
Supplement: Supplementary file 1 [file behavsci-15-01462-s001.zip › behavsci-3767843-supplementary.pdf]

behavioral sciences

MDPI

Supplementary Table S1. Proportion of endorsement of experiencing each Potentially Traumatic Event of the Life Events Checklist in Samples 1 and 2.

Event Type	Sample 1 (*n = 339), N (%)*	Sample 2 (*n = 318), N (%)*
Natural Disaster	87 (25.67)	94 (29.6)
Fire or Explosion	28 (8.26)	22 (6.9)
Transportation Accident	189 (55.8)	186 (58.5)
Serious accident at work, home or during recreation	58 (17.1)	58 (18.2)
Exposure to toxic substance	13 (3.8)	9 (2.8)
Physical Assault	67 (19.8)	84 (26.4)
Assault with a weapon	7 (2.1)	13 (4.1)
Sexual Assault	96 (28.3)	88 (27.7)
Other unwanted or uncomfortable sexual experience	197 (58.1)	170 (53.5)
Combat or warzone exposure	2 (0.60)	0
Captivity	5 (1.50)	2 (0.6)
Life-threatening illness or injury	33 (9.7)	39 (12.3)
Severe human suffering	10 (2.9)	6 (1.9)
Sudden violent death	21 (6.2)	13 (4.1)
Sudden accidental death	13 (3.8)	14 (4.4)
Serious injury, harm or death you caused to someone else	9 (2.7)	5 (1.6)
